# Autologous Intestinal Reconstruction Surgery in Short Bowel Syndrome: Which, When, and Why

**DOI:** 10.3389/fnut.2022.861093

**Published:** 2022-04-07

**Authors:** Giovanni Boroni, Filippo Parolini, Maria Vittoria Stern, Cristina Moglia, Daniele Alberti

**Affiliations:** ^1^Department of Paediatric Surgery, ASST Spedali Civili di Brescia, Brescia, Italy; ^2^Department of Paediatric Surgery, University of Brescia, Brescia, Italy

**Keywords:** short bowel syndrome (SBS), autologous intestinal reconstruction surgery, longitudinal intestinal lengthening and tailoring (LILT), serial transverse enteroplasty (STEP), spiral intestinal lengthening and tailoring (SILT), antiperistaltic reversed segment, colonic interposition, controlled tissue expansion

## Abstract

Short bowel syndrome (SBS), secondary to any natural loss or after any extensive bowel resection for congenital malformations or acquired disease, is the most common cause of intestinal failure in children. Extensive introduction of parenteral nutrition (PN) has dramatically changed the outcome of these patients, allowing for long-term survival. The main goal in children with SBS remains to be increasing enteral tolerance and weaning from PN support. Post resection intestinal adaptation allows for achievement of enteral autonomy in a subset of these patients, but the inability to progress in enteral tolerance exposes others to long-term complications of PN. Autologous intestinal reconstruction surgery (AIRS) can facilitate the fulfilment of enteral autonomy, maximizing the absorptive potential of the remaining gut. All the different intestinal reconstruction techniques, from simple procedures like tapering, reversed segments, and colon interposition, to more complex lengthening procedures (LILT: longitudinal intestinal lengthening and tailoring, STEP: serial transverse enteroplasty, and SILT: spiral intestinal lengthening and tailoring) and techniques designed for peculiar problems like controlled intestinal tissue expansion or duodenal lengthening are presented. AIRS indications, clinical applications, and results reported in the literature are reviewed.

## Introduction

Intestinal failure (IF) is defined as critical reduction in bowel mass or its function falling below the minimum level needed to absorb fluids and nutrients required for adequate growth in children and weight maintenance in adults (1). Short bowel syndrome (SBS) represents the most common condition that causes IF in children and is a consequence of massive reduction in bowel length secondary to surgical resection in patients with necrotizing enterocolitis, abdominal wall defects, bowel atresia, and midgut volvulus (2).

The introduction of parenteral nutrition (PN) in the late 1960s (3) dramatically improved the long-term survival of patients previously sentenced of a fatal outcome. Intestinal transplantation (4) has also introduced a chance of cure for patients with irreversible IF and PN-related complications such as IF-associated liver disease (IFALD), loss of central venous access, recurrent central line-associated bloodstream infections, and recurrent episodes of severe dehydration (5).

Care of children with SBS remains particularly challenging and requires the contribution of several specialists (surgeons, gastroenterologists, pediatricians, dieticians, social workers, pharmacists, and nurses) working in a multidisciplinary IF team (6). Main goals of treatment include normal growth and development, prevention of complications associated with SBS status and its treatment, and reduction of PN support until reaching enteral autonomy to fully exploit the adaptation potential of the remaining bowel (7). Autologous intestinal reconstruction surgery (AIRS) must be considered as an integral part of a structured plan to reach enteral autonomy.

## Consequences of Bowel Resection and Intestinal Adaptation

Extended intestinal surgical resection causes massive loss of mucosal surface, resulting in compromised absorptive capacity. Pathological consequences and the possibility of achieving enteral autonomy depend on both extension and site of resection. The length of the remaining small bowel (SB) is the single most important prognostic factor in prediction PN dependence; other factors include preservation of the ileocecal valve (ICV) and retained colon. Among neonates with 40–80 cm of residual SB and type 3 SBS (jejuno-ileocolic anastomosis with preserved ICV and colon), 80% achieved enteral autonomy within 1 year, whereas 40% of those with less than 40 cm of residual small bowel and without ICV remained dependent on PN after 8 years (7). In neonatal age, particularly in preterm infants, the percentage of expected SB length for gestational age is a more accurate predictor than the simple length of remnant SB. In the international literature, a residual SB length > 25% of that expected for gestational age is considered the minimum required for enteral autonomy (2), even if some authors report that 83% of children who retained ≥ 10% of expected small bowel length were able to wean off PN, compared to 10.5% of those with < 10% of expected small bowel length (8).

The remaining colon contributes to intestinal autonomy by absorbing water and electrolytes and metabolizing carbohydrates to short-chain fatty acids. The role historically recognized for the ICV is to slow the intestinal transit and impede the ascendance of colonic microbiota, preventing small bowel bacterial overgrowth (SBBO). However, most beneficial effects related to the retained ICV can be attributed to the residual ileum next to the valve. The ileum has a great adaptive potential, and it is the major source of intestinotrophic peptides like glucagon-like peptide 2 (GLP-2), which is probably the most powerful mediator of mucosal hypertrophy (7, 8).

Intestinal adaptation is a natural compensatory process that starts after extensive intestinal resection; it includes functional and structural changes in the residual SB, and has an aim of improving nutrient and fluid absorption.

Animal studies demonstrated many functional modifications after short bowel resection, such as increased expression of transporter proteins and exchangers involved in nutrient, electrolyte, and water absorption. Enterocytes express digestive enzymes and amino acid transporters more rapidly in the small bowel remnant after resection. Deceleration of small bowel transit after distal resection, which increases contact time between nutrients and the mucosa, is another mechanism for functional adaptation in experimental studies (7, 9). In animal models, small bowel resection is followed by acceleration of crypt cell proliferation with increase in crypt depth and villus height; intestinal resection is also associated with local angiogenesis and increase in tissue oxygenation. These structural changes cause mucosal growth and enhanced absorption. In humans, after resection, major morphological changes have been observed, with lengthening and dilatation of the remnant bowel and smooth muscle hypertrophy (9, 10).

The mentioned adaptive mechanisms occur with the aim of increasing absorption and gain enteral autonomy, but sometimes some of these, especially excessive dilatation, can determine pathological consequences. The dilated bowel does not have normal peristalsis, because the increased diameter impedes complete coaptation of the bowel walls, leading to sloshing motion of intraluminal contents and disorganized antegrade progression. These dilated segments, therefore, become sites of stasis and bacterial overgrowth, with mucosal inflammation, impaired absorption, high risk of bacterial translocation and sepsis (11).

Hukkinen et al. (12) recently showed that small bowel dilatation predicts prolonged PN duration and decreased survival in children with SBS. They calculated the ratio between the width of the largest short bowel segment and the height of the fifth lumbar vertebra. Patients with a ratio > 3 were 14.3 times less likely to wean off PN and had worse probability of survival than patients with a ratio < 2.

## Role of Non-Transplant Surgery

Surgical management of SBS begins with prevention: early recognition of high-risk situations for loss of critical bowel length, such as midgut volvulus and necrotizing enterocolitis, can ensure prompt and appropriate treatment. In case of bowel resection, every effort should be made to preserve as much bowel as possible, for instance maintaining even short segments between atretic tracts in multiple atresia or preserving bowel of questionable viability, in anticipation of a second look after 24–48 h. The ileocecal region must be preserved whenever possible. During the first operation, the anatomy and length of the remaining bowel should be recorded in detail for guidance of subsequent treatment (5, 13).

When an SBS has been established, the surgical priority is recruitment of all available bowels by dealing with pathologic situations such as blind loops, enterocutaneous or entero-enteric fistulas, and by taking down stomas. If temporary diversion is necessary, the recruitment of whole bowel may be accomplished by recycling of proximal stomal secretion in the distal intestine (7).

A multidisciplinary approach, in the context of an intestinal rehabilitation program, with optimized parenteral support and enteral nutrition, careful maintenance of central venous line, adoption of novel medical and hormonal therapies, such as the GLP-2 analogue teduglutide, allows most patients to reach enteral autonomy.

In patients dependent on PN, without any progression to enteral autonomy despite optimized medical and nutritional therapy, AIRS can be considered to improve intestinal absorption, and to correct pathological consequences of SBS and intestinal adaptation process (14).

The goals of AIRS procedures fall into four main surgical settings: to (a) slow down intestinal transit to increase contact time between nutrients and mucosa; (b) correct SB dilatation and stasis; (c) improve intestinal motility; (d) increase mucosal surface area.

Some procedures are designed to achieve one of these targets [e.g., reversed segments (RSs) only slow down intestinal transit], whereas other procedures can improve multiple aspects (lengthening procedures correct dilatation, ameliorate intestinal motility, and increase mucosal contact time) (Table 1). It is essential to understand that each procedure has its own indications and clinical applications, and that all surgical decisions must be tailored specifically to a single patient (2).

**TABLE 1:** Autologous intestinal reconstruction surgery (AIRS) procedure.

	Procedure	Goals*
Slowing procedures	Recirculating loop (15) Intestinal pouch (16) Intestinal valves (18–21) **Antiperistaltic reversed segments** (24, 26) **Colonic interposition** (42, 43)	T
Tapering procedures	**Tapering** (52) Plication (54)	D, M
SB lengthening procedures	**Longitudinal intestinal lengthening and tailoring** (55) Iowa model (60) Composite bowel loops (61) **Serial transverse enteroplasty** (73) **Spiral intestinal lengthening and tailoring** (100)	T, D, M, (A**)
SB expansion procedures	Nipple valve (105) **Controlled Tissue Expansion** (22) Delayed correction (108)	A
Duodenal lengthening procedures	Duodenal serial transverse enteroplasty (110) Iowa model (60) Transverse flap duodenoplasty (111)	T, D, M

*T, slow intestinal transit to increase contact time; D: correct dilatation; M: improve motility; A: increase mucosal surface area; **if some post-procedure dilatation occurs; SB: small bowel. Bold indicates the procedures most used and extensively treated in the article.

## Procedures to Slow Intestinal Transit

In patients with rapid transit time and with no bowel dilatation, procedures to slow down intestinal transit could be a viable way to enhance intestinal absorption and reduce parenteral support. Some of the earlier techniques, such as recirculating loop (15) and creation of intestinal pouch (16), have never gained widespread acceptance, and they are definitively abandoned (17).

Different techniques for creating artificial intestinal valves, as substitutes for the ICV, have been reported (18–21). The effect on intestinal motility involves various mechanisms: partial mechanical obstruction, disruption of normal motor pattern, and prevention of retrograde reflux of colonic content. However, the degree of obstruction is fixed and may be insufficient or, on the other hand, may lead to stasis in the proximal bowel, requiring valve resection. These limitations and inconsistency in the outcomes have widely limited the use of artificial valves in SBS treatment (17, 22).

### Antiperistaltic Reversed Segments

**FIGURE 1:**
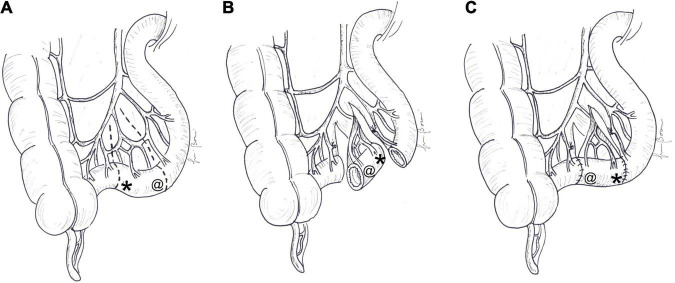
Antiperistaltic reversed segment: **(A)** a short segment of the small intestine is divided, **(B)** rotated by 180^°^, and **(C)** re-anastomosed, usually at the distal end of the jejunum. The symbol “*” indicates the original distal end and “@” the original proximal end of the reversed segment. At the end of the procedure, the proximal end (@) becomes distal and the distal end (*) becomes proximal.

An antiperistaltic RS is constructed by dividing a short segment of small intestine, rotating it by 180^°^, without impairing its blood supply, and re-anastomosing it, usually at the distal end of the jejunum, just proximal to an end-stoma or to the anastomosis with the colon (Figure 1). First experiments on RS in animals were reported by Mall (23), but the use of this technique on experimental models of SBS was introduced in the 1950s and 1960s. Many experimental studies with an RS have demonstrated prolongation of transit time, improved absorption, better nutritional status, weight gain, and prolonged survival (24–27), but other reports have shown no beneficial effects (28–30). Structural changes like dilatation of the bowel proximal to the RS and increase in muscle thickness, crypt depth, and villus height were described (24, 31). Electromyographic and manometric studies demonstrated that migrating a motor complex is often interrupted in the jejunum above the RS, and that myoelectrical activity in the segment is reversed and independent, maintaining the original polarity. The peristaltic wave in the RS passes proximally, causing delay in intestinal flow (29, 32, 33).

The first clinical application of the technique was reported in 1962 by Gibson et al. (34), and Fink and Olson, in (35), described for the first time a case in which two different RSs was placed in the same patient (35). Cywes 1968, published the first pediatric experience of RS in an 18-month-old boy (36).

The literature suggests a length between 10 and 15 cm in the adult population and 3 cm or more in children (17, 37); shorter segments may be ineffective in slowing intestinal transit, while longer segments may create a certain degree of bowel obstruction.

Some small series have been published in the literature, mostly on adults. Panis et al. (37), reported on eight patients with SBS (with median residual bowel length of 40 cm) who underwent a segmental reversal of 12 cm of small bowel. Three of the patients reached full enteral autonomy, 1 only had fluid and electrolyte infusion, and the four other patients reduced their parenteral support to four nights per week. The same authors, in 2012, published their experience in 38 patients, with a median length of the SB remnant of 49 cm and an RS of 10 cm (6–15 cm). In the 5-year follow-up, 45% of the patients were weaned from PN and, in the remaining patients, parenteral support was decreased from 7 ± 1 to 4 ± 1 days per week, with an overall survival rate of 84% (38). Concurrent ostomy takedown made it difficult to determine which procedure had greater responsibility for any clinical improvement. To support the predominant role of RS, 17 of these patients were matched to 17 patients with SBS patients, with the same digestive characteristics but without reversal of bowel segments. Patients with an antiperistaltic RS exhibited higher intestinal absorption of total calories, fats, and proteins, higher oral autonomy, and lower PN dependence (39). Thompson created an RS 10–15 cm in length for 16 cases among 520 patients with SBS. All the patients had a remnant bowel length of more than 80 cm and rapid intestinal transit. Nine (56%) improved, but 7 (44%) remained on PN or had persistent intractable diarrhea (40).

Nowadays, antiperistaltic RS is rarely employed, but in a selected subgroup of patients with rapid intestinal transit and adequate intestinal length with no dilatation, the procedure may be considered either in isolation or as a part of combined techniques (22, 41).

### Colonic Interposition

**FIGURE 2:**
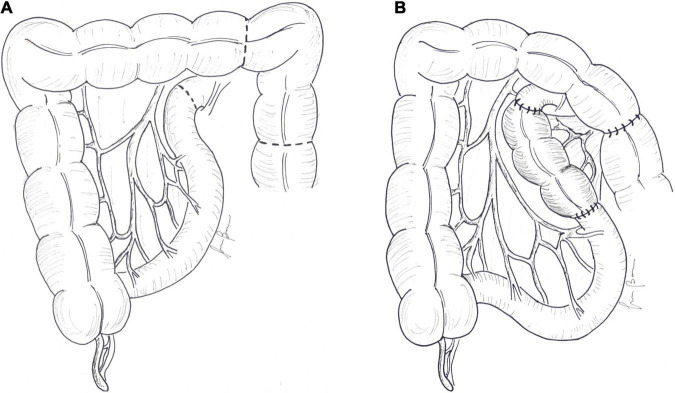
Colonic interposition: **(A)** 10–15 cm of the colon is divided and **(B)** interposed proximal to the remaining small bowel.

Peristaltic activity in the colon is different from that of the small intestine, being slower and segmental. Isoperistaltic or antiperistaltic interposition of a colonic segment proximal to the remaining small bowel (Figure 2) can slow transit time and, providing absorption of water and electrolytes, increases the concentration and viscosity of the chime entering the distal small bowel.

An initial description of the procedure has been reported by Hutcher et al. on an animal model of SBS: pre-jejunal and pre-ileal colonic transposition eliminated the 53% mortality seen in control animals and allowed for the attainment of 68% of expected growth (42, 43). Interestingly other experimental studies demonstrated morphological and functional changes in the interposed colon that reflected features of the small bowel: increase in crypt depth and mucosal thickness, rise in maltase levels, and better absorption of D-glucose, L-alanine, and L-phenylalanine (44–46). These adaptive changes have also been confirmed in humans: histological examination of an endoscopic biopsy specimen of the interposed colon revealed that the mucosa showed hypertrophy and hyperplasia of the crypt glands and cells resembling Paneth cells, which are usually seen in the small intestine (47).

Garcia et al. reported the first clinical description. A 24 cm isoperistaltic colonic segment was interposed in a 14.5-month-old boy after the procedure transit time was increased from 10 to 105 min and parenteral support was stopped (48). The largest available study included 6 infants who underwent isoperistaltic colon transposition (length 8–15 cm). Three of the infants were weaned from PN, but 3 others could not stop parenteral support and died of PN complications 11–26 months after the colonic interposition (49). Colonic transposition presents some theoretical advantages: viability of the small intestine is not compromised; residual colonic function is not impaired; the procedure is possible when the remaining small intestine is too short to permit reversal of the small bowel (50). Despite these benefits, the procedure has seen limited use, and only isolated cases are reported in the recent literature (51).

## Procedures to Correct Bowel Dilatation and Stasis

**FIGURE 3:**
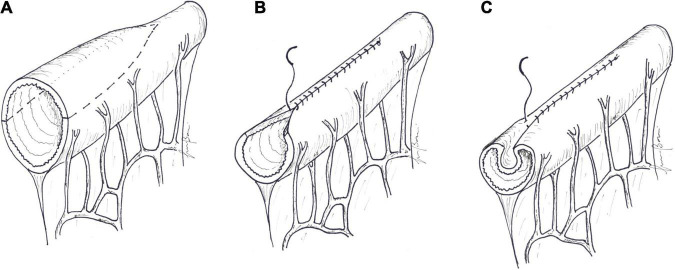
**(A)** In tapering, a wedge-shaped strip of the antimesenteric border is resected and **(B)** the remaining bowel is sutured into a tube. **(C)** In plication, the antimesenteric border of the dilated bowel is folded into the lumen, and the lateral margins are sutured.

As a result of the adaptation process, some patients develop SB dilatation that causes stasis and disturbance in propulsion and mixing of bowel contents. Bacterial overgrowth can develop in this situation, with consequent aggravation of malabsorption, mucosal injury, and epithelial permeability, and increased risk of sepsis. Excessive intestinal dilatation can be easily managed by simple tapering enteroplasty, in which a wedge-shaped strip of the antimesenteric border is resected and the remaining bowel is sutured into a tube. The procedure can be quickly accomplished with a stapler (52, 53) (Figure 3). The major drawback of tapering is waste of enterocyte mass. In order to avoid loss of absorptive mucosa, de Lorimier and Harrison (54) proposed plication of the antimesenteric border of dilated bowel (Figure 3). Despite initial success, sutures often opened up, which allowed for the bowel to return to its former dilated state. These procedures are effective in reducing stasis and improve intestinal motility, but the availability of lengthening techniques confines the use of tailoring and plication only to a small number of patients with SBS with sufficient absorptive bowel and isolated dilated intestinal loops.

## Lengthening Procedures

Correction of bowel dilatation without loss of the mucosal surface area is accomplished with lengthening techniques. In all these procedures different techniques are employed to reconfigure a widely dilated bowel to a narrower and longer one, achieving, in addition to bowel dilatation and stasis reversal, improvement in intestinal motility, slowing down of intestinal transit, and increase in contact time between nutrients and the mucosa. Moreover, some degree of redilatation, usually observed after lengthening procedures, eventually provides expansion of the surface area.

### Historical Notes

The description of the first lengthening technique, the longitudinal intestinal lengthening and tailoring (LILT) procedure, was reported by Bianchi (55).

In the early 1990s, Kimura and Soper developed in animal models the concept of isolated bowel segments. A seromuscular incision was made along the antimesenteric border of a jejunal segment, exposing the muscolaris mucosa. Corresponding incisions were made in a host organ: a sero-fascial incision on the undersurface of the abdominal wall in the Iowa model 1 (56, 57); an incision on the liver capsule in the Iowa model 2 (58); a seromuscular incision on a bowel segment in the Iowa model 3 (59). The deserosed antimesenteric surface of the jejunal segment was then sutured to the host organ. After a period of several weeks, the mesentery of the jejunal segment was divided, and the isolated bowel preserved its viability by vascular collaterals that originated from host organs during the interval between the two procedures. Kimura and Soper described the first clinical case in which the Iowa models were applied for a bowel elongation technique (60). A six-week-old boy had an ultra-short bowel due to intrauterine midgut volvulus, with the remnant bowel only represented by the dilated duodenum anastomosed to the distal colon. The authors created an hepato-myoenteropexy between the dilated duodenum and the anterior liver margin and the undersurface of the abdominal wall. After 16 weeks, the duodenum was divided horizontally, and two bowel loops were created: the first one from the “mesenteric” half of the duodenum, and the second one from the antimesenteric half, vascularized by blood supply derived from the liver and abdominal wall. The two loops were then anastomosed in an isoperistaltic fashion, creating a longer and narrower duodenum. At the age of 18 months, the child tolerated 50–60% of required calories *via* the enteral route. The complexity of this surgery, wait time required for parasitization to occur, and need for multiple laparotomies resulted in the abandonment of this technique.

Even the composite bowel loops described by Bianchi (61) have never gained widespread acceptance. In these loops, a vascularized seromuscular flap, isolated from the greater curvature of the stomach or from the colon, was applied to the exposed submucosa in the antimesenteric border of the dilated jejunum. After 6 weeks, a new blood supply developed across the graft interface; the dilated jejunum was divided horizontally, and two hemi-segments were tubularized and anastomosed to increase total intestinal length (61, 62).

In addition to LILT, the only two lengthening techniques that have been used in clinical practice are serial transverse enteroplasty (STEP) and the most recent spiral intestinal lengthening and tailoring (SILT).

### Longitudinal Intestinal Lengthening and Tailoring

The LILT technique is the first AIRS procedure designed to double the length of a loop of dilated small intestine while simultaneously reducing its luminal diameter and preserving the maximum amount of small bowel mucosa. LILT is based on the observation that mesenteric vessels, which are allocated alternately to one or the other side of the bowel loop, do not enter the bowel wall directly in the midline but rather to one or the other side, leaving a relatively avascular space along the mesenteric border. In the original description, peritoneal leaves of the mesentery were dissected apart, leaving each mesenteric vessels on its own side, and the anvil of a stapler was inserted in this avascular plane. The stapler was then closed, suturing the mesenteric and antimesenteric surfaces of the bowel and creating two intestinal loops of half the diameter (45). The use of staplers was replaced afterward by division of the bowel with bipolar diathermy and hand suturing (63). The procedure requires a minimal intestinal diameter ≥ 4 cm or at least a dilatation of double the normal SB size and a healthy mesentery.

**FIGURE 4:**
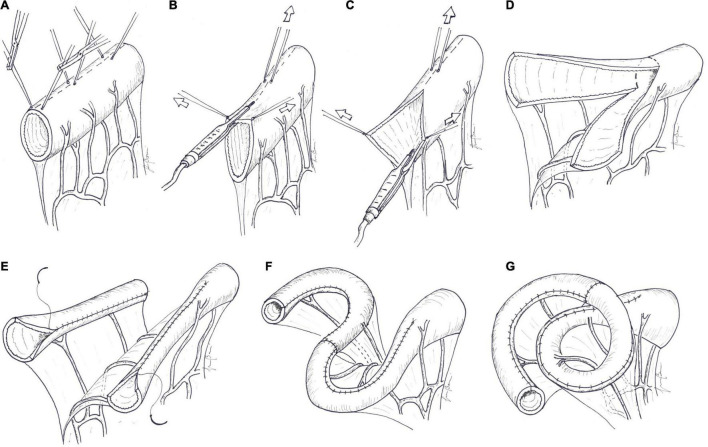
Longitudinal intestinal lengthening and tailoring. **(A)** A marker line is drawn longitudinally along the midline, in the mesenteric border of the dilated segment of the bowel, as an orientation guide. Traction sutures are placed along the antimesenteric border to the right and left of the midline, at about 5–10 cm intervals. **(B)** The antimesenteric border is divided longitudinally, passing between the traction sutures, by bipolar diathermy. **(C)** Outward and upward tractions of the opened bowel loop against the base of the mesentery gives access to the blood vessels between the two layers of the mesentery. The avascular space between the mesenteric vessel and the bowel wall is developed by blunt dissection. The mesenteric border is then divided longitudinally in the midline by cutting bipolar diathermy. **(D)** Longitudinal bowel division along the mesenteric border develops two vascularized hemi-segments. **(E)** One of hemi-segments is completely detached by division along the lateral wall and tubularized with a continuous suture. The other hemi-segment, in continuity with the proximal bowel, is tubularized in the same manner. **(F)** The two new hemi-loops are then anastomosed to each other in an isoperistaltic fashion. This can be accomplished in a “lazy S” shape or in a “spiral” shape **(G)**, as described by Aigrain.

Technique: in the initial stage of the procedure, encountered peritoneal adhesions must be released. The SB is carefully mobilized, with preservation of all mesenteric blood vessels. After that, bowel diameter and length are measured along the antimesenteric border. A marker line is then drawn longitudinally along the antimesenteric border of the dilated segment. Stay sutures are placed right and left of the midline, at about 5–10 cm intervals. By bipolar diathermy, the antimesenteric border of the bowel is thereby divided longitudinally, between the traction sutures. Outward and upward tractions of the opened bowel loop against the base of the mesentery give access to the blood vessels between the two layers of the mesentery. The avascular space between the mesenteric vessels and the bowel wall is developed by blunt dissection. The mesenteric border is then divided longitudinally in the midline by cutting bipolar diathermy, paying attention to not injure the mesenteric vessels. Longitudinal bowel division along the mesenteric border develops two vascularized hemi-segments. One of the hemi-segments is completely detached by division along the lateral wall, while the other is maintained in continuity with the proximal bowel. The 2 hemi-segments are tubularized with a continuous inverting Lambert suture of absorbable material, tying a securing knot every three or four throws. The two new hemi-loops are then anastomosed to each other in an isoperistaltic fashion. This can be accomplished in a “lazy S” shape, with the bowel lying over the mesentery, as described by Bianchi. In case of short mesentery, Aigrain et al. (64) proposed to anastomose the 2 hemi-loops in a “helix-like” or “spiral” shape, with one loop passing beneath the other (Figure 4). The distal end of the second hemi-loop is anastomosed to the distal bowel, often the colon, to establish bowel continuity.

Chanine and Ricketts described a technical variation focused on reducing the number of anastomoses: in their LILT modification, division of the intestine, performed with a surgical stapler, begins obliquely at the proximal end of the intestine to be lengthened, proceeds longitudinally as in the Bianchi procedure, and ends obliquely at the distal end of the intestine toward the opposing edge of the intestine to keep the ends in continuity with the bowel. The intestinal continuity is accomplished by a single anastomosis between the new created hemi-loops (65).

To avoid excessive traction on mesenteric vessels and nerves, “double barrel” enteroplasty has been recently described: the junction between the dilated proximal bowel and the normal-caliber distal bowel is transected, and the dilated bowel is stapled into two hemi-loops like in the original LILT procedure without dividing it from the proximal bowel. The two hemi-loops are left in parallel, and the double-barreled bowel is joined end-to end onto the distal bowel, or, if size discrepancy is significant, one hemi-loop is joined end-to-end and the other one end-to-side onto the distal bowel (66).

The first clinical application of LILT was reported in Boeckman and Traylor (67) on a 4-year-old boy with SBS secondary to vanishing gastroschisis. The small bowel remnant, anastomosed with the transverse colon, measured 50 cm and was greatly dilated in the distal 30 cm, with a diameter of 11 cm. This distal part underwent the LILT procedure; after 10 weeks, the patient reached full enteral autonomy, and parenteral support was stopped.

The first clinical series of 20 infants who underwent LILT was reported by Bianchi (68). Cholestasis was present in all the patients. The original bowel length varied between 25 and 98 cm, with the diameter of the dilated loop > 5 cm in all the cases. One child developed an enterocutaneous fistula that resolved spontaneously, whereas in 2 of the patients, a stenosis at the hemi-loop anastomosis required surgical revision. In a mean follow up of 6.4 years, overall survival was 45%. Of the 9 survivors, 7 progressed to full enteral autonomy and 2 required partial parenteral support. Cholestasis resolved spontaneously. In 10 of the 11 non-survivors, after an initial improvement, liver function worsened; eventually, they died from end-stage hepatic failure. For this reason, the author considered more appropriate to propose LILT in an early stage, before the onset of major hepatic dysfunction (68, 69).

In the series from the Children’s Hospital of Pittsburgh, the overall survival of 19 patients who underwent LILT was 79% (15 patients), but nine of the surviving patients required rescue by short bowel transplantation. Sixteen of the patients were successfully weaned from PN: 8 (42%) responded to LILT alone, and 8 had decreased TPN dependence but ultimately underwent transplantation (70).

Hosie et al. (71) reported the results of LILT performed on 49 children with mean age of 25 months (range of 4 months to 12 years). Preoperatively, the small bowel mean length of 27 cm (12–60 cm) was increased to 51 cm (18–120 cm). Ischemia of a 2-cm bowel segment, which needed to be resected was observed in 2 of the patients. Two of the patients developed leakage along the longitudinal suture, and 1 developed intra-abdominal abscess, necessitating re-laparotomy. Nineteen of the patients (39%) were weaned from PN, and 5 required some parenteral support at home. Nine (18%) of the patients died, mostly because of end-stage liver failure or sepsis. The most frequent encountered complication was recurrent dilatation of the lengthened bowel loops, with dysmotility, stasis, and bacterial overgrowth. Unfortunately, 16 (32%) patients were lost to follow-up, impeding an accurate analysis of the results.

Reinshagen et al. (72) reported a large series of 53 patients. In a median follow up of more than 6 years, 41 of the patients (77.3%) survived, and 36 (68%) were successfully weaned from PN. Length of the residual SB together with length of the colon and preoperative liver function were reported as prognostic factors for survival after LILT.

### Serial Transverse Enteroplasty

In serial transverse enteroplasty (STEP), lengthening of the dilated bowel is performed by serial transverse application of a GIA stapler from opposite direction to create a zig-zag channel of approximately 2 cm in diameter. The procedure is based on the anatomic principle that blood supply traverses the bowel remaining perpendicular to the long axis of the bowel; if staple lines are kept perpendicular to the long axis, all segments should remain well-vascularized.

Kim et al. proposed this novel bowel lengthening procedure (73) in animal models. After STEP, all the animals gained weight and showed no clinical or radiological evidence of obstruction. At the end of the experiment, six weeks after surgery, the lengthened segment had become practically straight, and gain in length was 64 ± 25%. Interestingly the STEP channel size increased from 2.1 to 4.3 cm, while the distal control bowel increased from 3.6 to 3.8 cm. The same group examined the effects of STEP on intestinal absorption and motility in animal models of SBS. The STEP animals, compared with controls, showed improved weight retention, increased intestinal carbohydrate and fat absorption, and elevated levels of serum citrulline, a marker of intestinal mucosal mass (74). Manometric and strain gauge monitoring demonstrated no difference between the STEP animals and the controls for presence and characteristics of phase III of the migrating motor complex (MMC) (75). Using a rodent model, Kaji et al. demonstrated that the STEP procedure had a significant effect on intestinal morphology: there was not only increase in bowel length but also significant increase in villus height, decrease in crypt apoptosis, and rise in post-prandial production of GLP-2 (76). Piper et al. proved the feasibility of a repeat STEP operation in a pig model (77). Instead of a GIA stapler, use of radiofrequency energy to perform STEP was successfully applied in animals but never described in humans (78).

**FIGURE 5:**
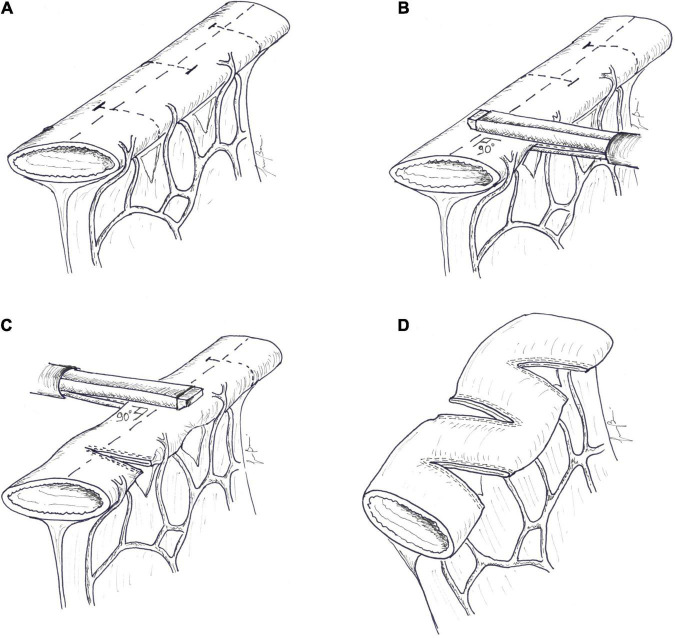
Serial transverse enteroplasty (STEP). **(A)** A marker line is drawn longitudinally along the midline, in the antimesenteric border of the dilated segment of the bowel, as an orientation guide. A small defect is created in the mesentery. **(B)** The larger side of an endoscopic GIA stapler is inserted perpendicular to the long axis of the bowel. The bowel is flattened, keeping the antimesenteric marker line strictly in the midline, and the stapler is applied; **(C)** The next cut is taken distally to the previous cut, and the stapler is now inserted from the opposite side. The procedure is carried on serially, until all the dilated bowel has been treated. **(D)** The final result is a zig-zag lengthened bowel. Channel size, usually about 2 cm, is determined by the length of the staple-line and the distance between stapler applications.

Technique: at the beginning of the procedure, bowel diameter and length are recorded. A marker line is then drawn longitudinally along the antimesenteric border of the dilated segment of the bowel. A small defect is created in the mesentery for the passage of a laparoscopic GIA stapler, which is inserted perpendicular to the mesentery and to the long axis of the bowel. An 18-F red rubber catheter can be passed through the defect as a guide for the larger side of the stapler. In this way, the marker of the end of the staple line, printed on the smaller jaw of the stapler, is clearly visible (79). The bowel is flattened keeping the antimesenteric marker line strictly in the midline, and the stapler is fired. A 2-cm tract is left uncut between the end of the stapler and the opposite bowel border, which will represent the final diameter of the reconstituted bowel loop. The next cut is taken about 2 cm distally to the previous one, and the stapler is now inserted from the opposite side. The procedure is carried on serially until all the dilated bowel has been treated. A suture is placed in the staple line apex to reduce the risk of leak from this area. The final result is a zig-zag lengthened bowel. The total theoretical increase in length depends on the degree of bowel dilatation and the size of channel created; channel size, usually about 2 cm and variable based on the age and caliber of normal bowel, is determined by the length of the staple-line and the distance between stapler applications and can be tailored by surgeons (Figure 5).

The first clinical application of STEP was reported by Kim et al. (79) on a 2-year-old boy with SBS due to gastroschisis who had previously undergone a LILT procedure. Two years later, the same group reported successful short-term outcomes of STEP in 5 infants with SBS (80). In the same year, the technique was adopted as primary therapy in a neonate with proximal jejunal atresia but without SBS to address size discrepancy between the proximal and distal segments (81).

In 2007, Wales et al. published the medium-term outcomes of 14 patients who underwent STEP, with mean increase in length of the dilated bowel of 94 ± 30% and increase in total small bowel length of 49 ± 42%. Three of the patients experienced complications: 2 staple line leaks and 1 gastrointestinal bleeding from staple line ulcers. Three of the patients died, and 2 received combined small bowel and liver transplantation. Seven of the patients were weaned from PN (82) and, in a five-year follow-up, they remained free from PN, with steady weight around the 35th percentile and with no long-term complications reported (83). In a single-center series of 20 STEP procedures, tolerance for enteral nutrition increased from 22% 1 month pre-STEP to 61% 6 months post-STEP; more specifically, enteral nutrition increased in 75% of the patients, with 25% of the patients achieving enteral autonomy, while 25% had unchanged or decreased enteral nutrition (84). A systematic review focused on enteral tolerance following STEP showed that 87% of 86 children who underwent STEP had increase in enteral tolerance (85).

Soon after the introduction of the STEP procedure, the International STEP Data Registry was instituted to allow for larger, multicenter patient accrual and follow-up. At present, 2 reports have been published in the literature from the Registry. The first one, in 2007, analyzed indications, efficacy, and complications of the first 38 patients enrolled (86). Indications for STEP were SBS with dependence on PN in 29, bacterial overgrowth in 6, and neonatal atresia with marginal residual bowel length in 3. Mean intestinal length increased from 68 ± 44 to 115 ± 87 cm, with a relative 69% increase in overall small bowel length. Among patients with dependence on PN, in a median follow up of about 1 year, there was an overall post-STEP rise in enteral tolerance by116%. Of the 6 patients operated for bacterial overgrowth, 5 had complete resolution of their symptoms. Complications related to the STEP procedure included intraoperative staple line leak (2 patients), bowel obstruction (2 patients), and fluid collection or abscess (3 patients). Three of the patients (7.9%) died because of progressive liver disease and sepsis, and 3 of the patients required transplantation. The most recent report from the International STEP Data Registry (87), reported the results of 97 of the 111 patients enrolled. Eleven of the patients (11.3%) died, and 5 (5.1%) progressed to intestinal transplantation. Higher direct bilirubin and shorter pre-STEP bowel length were predictive of these events. Of the 87 transplant-free survivors, 48 (55%) were weaned from PN, 14 (16%) had increased enteral tolerance, 8 (9%) had no change or reduced enteral tolerance, and 14 underwent a second STEP procedure.

The first report on successful application of a second STEP procedure to further lengthen the small bowel in SBS was reported in 2007 (88). Andres et al. published the results of redo-STEP on 14 patients after prior LILT (*n* = 7) and prior STEP (*n* = 7) (89). Survival was 100%. After the redo-STEP, discontinuation of PN was achieved in 6 (43%) of the patients, while intestinal transplantation was performed on 4 (28.5%). These results were not confirmed by other authors. In a report from the International STEP Data Registry, only 3 (21%) of 14 patients that underwent redo-STEP were weaned from PN (87). Barret et al. reported a greater increase in enteral nutrition after first STEP compared to redo-STEP (26 vs. 4.7%), and no patients reached enteral autonomy after redo-STEP (90). Mucosal inflammation seemed to be correlated with persisting bowel dysfunction after STEP and need for re-STEP, especially in the absence of the ICV (91).

Serial transverse enteroplasty is a quite easy procedure to perform, as there are no anastomoses, the bowel is never opened, and the mesentery is not jeopardized by any dissection. The degree of tapering is customizable and, with massively dilated bowel, it is possible to more than double the length of the bowel. Lastly, STEP can be performed after a LILT or STEP procedure if redilatation occurs.

### Longitudinal Intestinal Lengthening and Tailoring vs. Serial Transverse Enteroplasty

Longitudinal intestinal lengthening and tailoring and STEP are main AIRS techniques used today and are the only ones for which a sufficient number of publications are available. In the literature, there are only few single-center series (92–94) and two systematic reviews (95, 96) comparing LILT and STEP. When analyzing the results of these studies, one must bear in mind the following issues: (a) some authors prefer LILT as initial procedure and use STEP when LILT is not applicable because of mesenteric vascular configuration and/or foreshortened mesentery (92, 94); (b) LILT is often used with worse results, notably in the first years after its description, in patients with advanced liver disease, which is now a contraindication for any type of AIRS (68); (c) STEP is sometimes performed as primary therapy in neonates with congenital dilated bowel (e.g., proximal jejunal atresia) but without SBS (81); (d) the longer follow-up for patients who underwent LILT may affect the rate of long-term complications and enteral autonomy; (e) the survival rate of procedures applied in different periods (from the 1980s for LILT and after 2003 for STEP) may reflect not only the benefits of the procedure but also the progress in medical treatment of patients and introduction of a multidisciplinary approach to SBS.

In a single-center experience reported by Sudan et al. (92), 64 patients, including 14 adults, underwent 43 LILT and 21 STEP procedures between 1982 and 2007. The overall survival was 91% (LILT 88%, STEP 95%), with no difference in survival based on which the lengthening procedure was performed. Fifty-eight percent of the patients were weaned from PN, and there was a trend toward an increased rate of weaning in the patients who underwent STEP (60%) compared with those who underwent LILT (55%). Although intestinal transplantation was performed more commonly after LILT (18.6%) than STEP (5%), this may be because of the shorter follow-up of the patients who underwent STEP. An early major postoperative complication occurred in 10% of the patients, and the incidence did not differ between the two procedures. Recurrent bowel dilatation, treated by re-STEP, was reported in about 20% of the patients in both groups. Another recent single institution study (94) reported on 22 patients who underwent lengthening procedures from 2004 until 2014: 10 (45%) underwent LILT, 11 (50%) underwent STEP, and 1 (5%) underwent simultaneous LILT and STEP procedure. Twelve of the patients had a secondary lengthening procedure (STEP), and 2 had a third STEP. Only 1 of the patients underwent intestinal transplantation. Eleven (50%) of the patients were weaned from PN at the completion of the study; after the first lengthening procedure, 7 (32%) of the patients reached enteral autonomy (5/9 LILT, 1/7 STEP, and 1/1 combined LILT/STEP). No surgical complications were reported.

In 2013, two different systematic reviews on the LILT and STEP procedures were published: the results of these reviews are summarized in Table 2 (95, 96). Mortality and small bowel transplantation rates were higher for the LILT group, but this may reflect the different periods of application of the two techniques. King et al. actually demonstrated no significant difference in survival between LILT carried out since 1996 and STEP (96). The rate of weaning from PN was significantly higher for LILT procedures than for STEP. Here, it is also possible that the shorter length of follow-up for STEP patients has affected these results. A higher rate of complications was reported in STEP patients. LILT and STEP had similar stricture (LILT 17.7% and STEP 17.5%) and leakage (LILT13.2% and STEP 12.1%) rates. In contrast, bleeding occurred more frequently after STEP (22.2%) than after LILT (16.1%). Postoperative intestinal redilatation was reported on 39% of the cases after LILT and on 49% of the patients after STEP, and it was considered a serious complication that denotes the return of the bowel to a dysfunctional status and an indicator of poor outcome (93). Some surgical complications, such as intestinal necrosis (10.6%), perforation (10.1%), and fistulization (7.4%), were only reported for LILT.

**TABLE 2:** Summary of the results of systematic reviews on longitudinal intestinal lengthening and tailoring (LILT) and serial transverse enteroplasty (STEP).

		Frongia et al. (95)	King et al. (96)	
Articles reviewed		39 articles	28 articles	
Patients analyzed		363 LILT/109 STEP	276 LILT/127 STEP	
Lengthening	LILT	48% (25–100%)		
	STEP	63% (40–120%)		
Survival	LILT	69.8% (33.3–85.7%)	81%	*p* < 0.001
	STEP	85.7% (78.6–100%)	89%	
Weaning from PN	LILT	71.5% (4–100%)	54.9%	*p* < 0.001
	STEP	58.1% (20–100%)	47.9%	
SBTX	LILT	26.0% (5–52%)	9.9%	*p* = 0.002
	STEP	16.1% (7.9–25%)	6.2%	
Redilatation	LILT	39% (8–100%)	4.2%	
	STEP	49% (30–67%)	12%	
Complications	LILT		17%	*p* = 0.02
	STEP		26%	

*SBTX, small bowel transplantation.*

Although LILT and STEP are one-stage procedures that combine the benefits of reducing the diameter of dilated bowel and slowing transit time with preservation of enterocyte mass, some relevant differences exist between the 2 techniques. LILT needs a healthy mesentery with no fibrosis and a good vascular configuration; it is a surgically demanding procedure, and one unique complication that can develop following LILT is necrosis of one of the hemi-loops because of vascular compromise (14). LILT has a fixed degree of tailoring and can only reduce the diameter to half and double the length but do not alter the orientation of muscle fibers. STEP is an easier and more adjustable procedure, and theoretically can increase the length by more than 100%. The manipulation and dissection of mesentery is minimal, which reduce the chance of vascular compromise. Nevertheless, with STEP, there is disarrangement of muscle fibers; circular fibers become longitudinal, while longitudinal fibers become circular. It is not clear whether peristalsis is restored in the STEP segment or the intestine becomes passive (97, 98). If the operated segment redilates, STEP is repeatable on children who have undergone prior STEP or LILT. In contrast, LILT cannot be repeated on the same segment once a LILT procedure has been previously performed, but it is feasible after prior STEP (99).

### Spiral Intestinal Lengthening and Tailoring

Spiral intestinal lengthening and tailoring, the newest lengthening technique, was proposed by Cserni et al. (100) to overcome the abovementioned constraints of LILT and STEP.

In SILT, full-thickness spiral cuts are created on the dilated bowel, which is then stretched and retubularized. Initially, the procedure was developed using a bowel surrogate. After marking the orientation of muscle fibers, the wall of the intestinal simulator was cut into a spiral shape. Using an angle of 45°, the simulator was lengthened by 60%, and the diameter was tailored by 33%; 73% lengthening and 44% tailoring were achieved by spiral cut at 60°. The procedure was then adapted for porcine small bowel *ex vivo* that has lengthened by 136 ± 21%, and the diameter was reduced by 56 ± 8%. Microcirculation of the mucosa after SILT was also assessed *in vivo* with the intravital orthogonal polarization spectral imaging technique: the velocity of circulating red blood cells remained relatively high, and oscillation of the capillary flow, which is considered a sign of insufficient microcirculation, was not observed (100). In the first *in vivo* study, the same author reported the results of 6 Vietnamese mini pigs that underwent SILT with a cut angle of 45–60%. Mean lengthening was 74.8 ± 29.5%, and mean diameter reduction was 56.25 ± 18.8%. No necrosis, perforation, suture break, or peritonitis was observed. In 2 of 6 the animals, in which the lumen was narrowed by more than 70% to a diameter of less than 1.5 cm, bowel obstruction developed, and the autopsies revealed severe angulation of the SILT segment. The other 4 animals recovered uneventfully; after 5 weeks, all the lengthened segments were viable and showed peristalsis. On histological examination, the mucosa looked viable and slightly hypertrophic. No villus and crypt atrophy, infarction, inflammation or submucosal hemorrhage were detected. The orientation of muscle layers remained very similar to normal, and submucosus and myenteric ganglia appeared normal (98).

**FIGURE 6:**
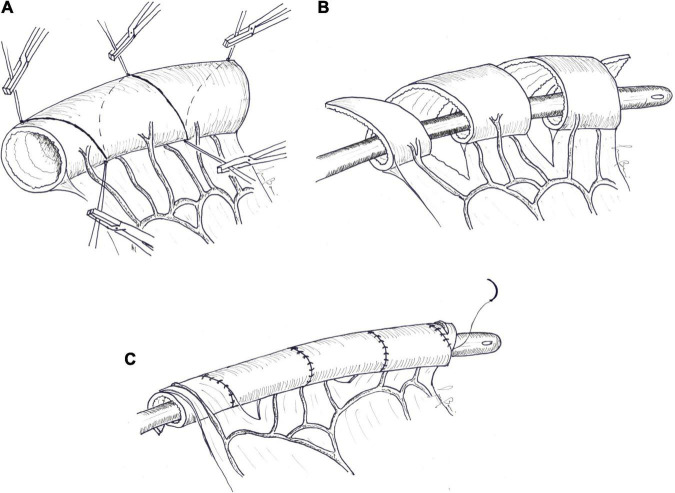
Spiral intestinal lengthening and tailoring (SILT). **(A)** Spiral incision lines are drawn at 45–60°. Stay sutures are placed where the spiral lines met on the antimesenteric and mesenteric borders. **(B)** The bowel is then cut into a spiral shape by bipolar diathermy following the previously drawn line. Once the spiral incision reaches the mesenteric border, the mesentery is also incised perpendicular to the longitudinal axis. The incised bowel is stretched longitudinally over an intraluminal silicon catheter. **(C)** Contiguous bowel edges are then sutured with an absorbable continuous introverting suture.

Technique: as for other AIRS techniques, the surgical procedure starts with meticulous adhesiolysis and thorough evaluation of the entire remaining bowel, whose diameter and length, along the antimesenteric border, are measured and recorded. Spiral incision lines are drawn at 45–60°. Stay sutures of different colors are placed where spiral lines meet on the antimesenteric and mesenteric borders. The bowel is then cut into a spiral shape by bipolar diathermy and following the previously drawn line. Once the spiral incision reaches the mesenteric border, the mesentery is also incised perpendicular to the longitudinal axis, staying in the avascular area between vasa recta and preserving the vascular arcades. The incised bowel is stretched longitudinally over an intraluminal silicon catheter to a uniformly longer tube of a narrower diameter. Interrupted absorbable sutures are placed every 3 to 4 cm to maintain the reconstructed shape. Contiguous bowel edges are then sutured with an absorbable continuous introverting suture, placing a knot after every third stitch (Figure 6). The distal end of the tailored segment is then anastomosed to the distal bowel in order to establish bowel continuity. If necessary, mesenteric defects are narrowed to prevent intra-abdominal herniation. Maximum achievable lengthening depends on anatomical factors, helix incision angle (45 to 60°), and number of coils. Cserni created a mathematic formula (α ≥ 90-arc sin R2/R1), which describes the relationship of the angle of the spiral cut (α) and the original (R1) and the desired final (R2) radii of the bowel, allowing surgeons to determine the best incision angle (100). Recently, a more complex mathematical model was described to help surgeons in defining optimal parameters for the intervention. With this model, knowing the length and the diameter of bowel that undergoes the SILT procedure, it is possible to set up the cutting angle to achieve the longest final length by guarantying the desired intestinal diameter and a manageable number of coils to be cut and sutured (101).

A modification of the SILT, leaving the mucosal layer intact during the procedure, was described and proved to be technically and clinically feasible on a large animal model (102), but no other reports on this modification were published.

The first clinical application of the SILT procedure was reported by Cserni et al. (103): a 3-year-old girl remained with only 15 cm of proximal jejunum after midgut volvulus; the rest of the small bowel and a part of the ascending colon were resected. After 12 months of controlled bowel expansion, the remnant bowel increased from 15 to 22 cm, and the distal 11 cm reached a diameter of 4 cm. This segment was lengthened by up to 20 cm by SILT to obtain a total jejunal length of 31 cm, with a diameter of 2 cm. No complications were reported. After 6 months, the patient tolerated 100% of calories by oral diet and overnight gastrostomy feeding. In the same year, Alberti et al. (104) published a second case report. A full-term neonate male was admitted for midgut volvulus and, after resection of necrotic bowel, only 4 cm of the jejunum and 5 cm of the distal ileum with the ICV and the entire colon remained. After controlled bowel expansion, the proximal jejunum elongated to 9 cm, with a diameter < 4 cm. The child underwent SILT with jejunal lengthening from 9 to 14 cm and a diameter reduction of 2 cm. The lengthened jejunum was anastomosed to the residual 5 cm of the ileum to give a total short bowel length of 19 cm. On the 8th postoperative day, the child developed an enterocutaneous fistula that closed spontaneously. In the 12-month follow-up, he maintained a steady growth and took more than 80% of required calories with normal oral diet.

The largest series of SILT cases, with a median follow up of 26 months, was reported in 2019 (97). Five children with SBS underwent SILT at the median age of 8.3 months. Preoperative small bowel diameter measured a median of 4 cm (3.5–4.6 cm). SILT allowed for a median increase in length of 56% and a reduction in diameter of 50%. None of the patients developed leak from the suture line or the stricture and small bowel obstruction, or required SILT-related further surgical intervention. One of the patients developed surgical site infection, which was treated conservatively with antibiotics, and 1 developed recurrent episodes of D-lactic acidosis. After 6 months, the need for PN support was reduced from a median of 7 to 4 nights per week. In this series, 3 of 5 patients received SILT in combination with other AIRS procedures (jejunal STEP in 1 case and tailoring of dilated duodenum on the antimesenteric border using serial stapler in 2 cases), impeding to determine whether the effects on weaning off parenteral requirements are solely due to SILT.

The SILT procedure has undoubtedly some advantages over LILT and STEP: it requires minimal handling of mesentery, determines minimal modification of muscle fiber orientation, and can be performed even in intestinal segments with fewer dilatations. In clinical practice, lengthening does not reach the 100% obtained with LILT, but in the cited series, the medium increase in length for the “SILTed” segments was 69% (97). Because of limited data, it is not possible to accurately determine the clinical significance of this procedure, but in selected situations it could be a valid complement in AIRS.

## Procedures to Increase Mucosal Surface Area

Although bowel dilatation can occur spontaneously during the intestinal adaptation phase, there are some patients who do not present any SB dilatation. In order to overcome the problem of patients with refractory SBS whose small bowel shows no tendency to dilate, and for whom no lengthening procedures can be offered, Georgeson et al. (105) suggested to surgically induce an SB dilatation that permits to do a subsequent intestinal lengthening procedure. A nipple valve was fashioned in the distal SB to provide temporary partial obstruction and to induce dilatation of the proximal small intestine. After 3–9 months, a LILT procedure was performed. Of the 6 patients treated with this sequential lengthening procedure, 1 reached enteral autonomy, 4 increased their enteral caloric intake from less than 10–50%, and 1 died 13 months later because of causes unrelated to the lengthening procedure. The same group demonstrated, with an animal model, that partial intestinal obstruction results in increase in mucosal thickness, villus height, crypt depth, and villus density. In other words, proximal dilatation results from transmural intestinal growth with creation of new absorptive mucosa and not simply from stretching of the existing mass (106).

Based these research studies, Bianchi introduced the concept of “controlled tissue expansion” (22). In initial laparotomy, when a short bowel state with no intestinal dilatation was discovered, a tube is passed into the proximal and distal bowel ends and brought out onto the abdominal wall as a tube stoma. At the time of feeding, the proximal tube is clamped for an increasing period of time, and unclamped before each feed. During expansion, the proximal stoma effluent is recycled into the distal tube, avoiding enteral deprivation and enhancing further absorption of water and nutrients. At the end of the period of tissue expansion, the patient undergoes a lengthening procedure. In 2011, first results on 10 patients treated by controlled tissue expansion were published (107). Initial bowel length was < 30 cm in all the patients. After 20–24 weeks of expansion, the initial SB circumference doubled, and there was a mean increase in length of 17.5%. All the patients underwent a LILT procedure, and 9 were subsequently weaned off PN.

Controlled tissue expansion is specifically designed to increase absorptive mucosal surface area and create additional tissues (mainly in circumference but also in length) to prepare patients for bowel lengthening and is not a solution in isolation. This procedure addresses some critical issues of nipple valve: with a tube stoma, the sacrifice of a segment of small bowel to create the valve is not required, and there is not a predetermined fixed degree of bowel obstruction, which may be either ineffective or excessive, but a system that allows for dynamic variation (22). Tissue expansion is not only reserved for children who fail to adapt and do not develop bowel dilatation but may be a primary indication for children who have undergone a catastrophic gastrointestinal event and are left with ultra-short bowel (small bowel length < 40 cm or < 20% of the expected length in preterm neonates).

For the same purpose, for ultra-short bowel due to vanishing gastroschisis and type IIIa jejunal atresia, some authors proposed to delay surgical correction after a period of induced intestinal dilatation (108). They reported on five patients in which, at the time of primary surgery, the proximal obstructed jejunum was not corrected and only a gastrostomy tube was placed. Sham feeds were then started by intermittent gastrostomy-tube clamping to induce bowel dilatation; after a median period of 108 days (range 27–232), STEP was performed, and continuity was established by the colonic remnant. In a median follow-up of 20 months, two of the patients were completely off PN, and two achieved > 50% enteral calories. Inability to properly drain the proximal jejunal pouch and exclusion of distal bowel from intestinal transit for a long time are the main drawbacks of this procedure.

## Duodenal Lengthening

In patients with SBS, it is not infrequent to find a dilated duodenum in continuity with the remnant dilated small bowel. Duodenal dilatation can interfere with the adaptation process facilitating alkaline reflux, dysmotility with stasis, and bacterial overgrowth with mucosa inflammation that impairs digestion and nutrient absorption. Recurrent episodes of symptomatic D-lactic acidosis are also reported (109, 110). The proximity of the pancreas, common blood supply with short vessels and absence of mesentery, close relationships with the common bile duct and pancreatic duct do not allow for LILT or SILT. In order to preserve as much mucosal mass as possible, a STEP procedure, with some technical modifications, has been proposed instead of conventional tapering, but only very few cases are reported in the literature.

Modi et al. described an 18-year-old woman with only 32 cm of residual bowel from pylorus to entero-colic anastomosis and recurrent episodes of D-lactic acidosis despite maximal medical treatment. The patient underwent duodenal STEP, and after 11 months of follow-up, only one episode of D-lactic acidosis occurred (110). Bueno et al. (109) reported three additional cases of duodenal lengthening in which a STEP procedure was applied. According to the author, the effect of tapering on the dilated duodenum improved intestinal motility, reduced stasis, and added extra intestinal length. Two patients achieved enteral autonomy after the procedure. Particularly, in a patient with only 5 cm of jejunal remnant and huge dilatation of the duodenum, STEP allowed to reach a total length of the bowel, from the pylorus to the colon, of 40 cm and to stop parenteral support (109).

In 1993, a single case of duodenal elongation was described by Kimura using Iowa models (60). Recently, a new procedure named “transverse flap duodenoplasty” was proposed for remodeling of dilated dysmotile duodenum (111). This procedure offers some advantages over STEP: there is no dissection on the pancreatic border that was kept intact; as such, there is no risk of injury to duodenal and pancreatic vessels, the easily identifiable ampulla of Vater, or to the bile duct and the pancreas. Moreover, the uniform construction avoids formation of diverticula that are a potential site for stasis and bacterial overgrowth. The experience, at the moment, is limited to 1 patient with a short follow-up.

## Conclusion

Short bowel syndrome is the most common cause of IF in children and is a consequence of natural loss or extensive SB surgical resection due to congenital malformations (gastroschisis, intestinal atresia, and total aganglionosis) and post-natal-acquired disease (necrotizing enterocolitis and midgut volvulus).

The estimated incidence in children is 24.5 per 100,000 live births, with a higher incidence reported in infants (95). Resection leads to impaired nutrient and fluid absorption, and patients become dependent on PN until intestinal adaptation occurs. The spontaneous intestinal adaptation process begins within 24–48 h after massive bowel resection and advances during the initial 6–24 months (9). Structural and functional changes observed in intestinal adaptation following resection result in gradual increase in absorptive capacity of the remaining bowel. The possibility of achieving enteral autonomy remains < 75% and is dependent on length, anatomy, and functional state of the remnant intestine as well as the underlying etiology (112).

In some patients, structural modifications induced by resection result in pathological consequences. Particularly, bowel dilatation leads to dysmotility and stasis, which promote bacterial overgrowth. SBBO can cause mucosal inflammation, impaired absorption, bacterial translocation with systemic infection, eventually contributing to the development of a liver disease that negatively impacts the ability to gain enteral autonomy and has a huge impact on the quality and expectancy of life of patients.

The survival of children with IF has substantially improved over the past 30 years. Increase in enteral tolerance (from reduction up to weaning off PN support) is the most important prognostic factor, by preventing PN-associated liver diseases and central line complications that remain the main indications for small bowel transplantation (7). Multidisciplinary intestinal rehabilitation programs have a key role in promoting intestinal autonomy, favoring the coordination of surgical, medical, and nutritional management.

Once a patient has reached the plateau of the advancement of enteral nutrition, despite optimal medical and nutritional treatments, two “types” of surgery could be considered: AIRS and small bowel transplantation. According to the most recent International Intestinal Transplant Registry, since 2000, actuarial patient survival has been 77% at 1 year and 58% at 5 years (113). Even if pediatric recipients are treated in high volume, experienced centers reach a survival rate of 87–92% at 1 year and 79–83% at 3 years, these results cannot be matched with the survival rate of 90–95% ensured by some multidisciplinary intestinal rehabilitation programs (114). Therefore, small bowel transplantation remains to be a salvage treatment for patients with irreversible IF who develop life-threatening complications such as liver failure, loss of central venous access, recurrent central line-associated bloodstream infections, and recurrent episodes of severe dehydration.

The chance to be weaned from PN after LILT and STEP is from 54.9 to 71.5% for LILT and 47.9 and 58.1% for STEP [Frongia et al. (95); King et al. (96)]. A recent review, on behalf of the Italian Society of Gastroenterology, Hepatology and Nutrition (SIGENP), investigated the role of AIRS in weaning from PN and reported that the prevalence of enteral autonomy was significantly higher in patients treated with PN alone (61.6%) than in patients receiving any AIRS (46.2%). Survival in the two groups was 91.5% for the PN patients and 95% for the AIRS patients (*p* = 0.82), and the incidence of liver disease was significantly higher in the patients treated with PN alone than in the AIRS patients (30.4% vs. 12%; *p* = 0.001) (112). One limitation of this review is that the two groups are not fully comparable. Because an indication for AIRS is impossibility to increase enteral tolerance, AIRS patients could have had more severe IF, even if the mean intestinal length was comparable in the two groups. The authors concluded that AIRS may be useful in selected patients.

There is broad agreement that AIRS is not appropriate for all patients, and that each procedure has its own indications and clinical applications and should be tailored to a single patient (2). Surgeon must be confident with every procedure in order to adapt surgery to the anatomic situation encountered during laparotomy. The main indication for AIRS is inability to reach enteral autonomy or progress to enteral tolerance despite optimal medical and nutritional treatments, particularly if bowel dilatation with SBBO occurs. It is mandatory to wait for a sufficient period to facilitate adaptation to the best possible level (7, 14, 95, 112). Other authors suggest a proactive strategy for patients with ultra-short bowel, and with anticipated low probability to reach enteral autonomy: in these patients, a controlled tissue expansion procedure may increase the absorptive mucosal surface area and create additional tissues to prepare the patients for bowel lengthening (13, 107). End-stage liver disease is associated with worse outcomes after lengthening procedures and is generally accepted as a contraindication for AIRS. In contrast, the presence of early signs of PN-associated liver disease may be corrected after AIRS and should be considered, in the presence of intestinal dilatation, as an inclusion criterion.

When a patient is considered for AIRS, the first step is to correct every situation that can impair the possibility to advance to enteral tolerance: all available bowels must be recruited by dealing with pathologic situations such as blind loops, enterocutaneous, and entero-enteric fistulas, and by taking-down stomas. Lengthening procedures are the most frequently adopted, because they can achieve correction of bowel dilatation and stasis, improvement of intestinal motility, and increase contact time between nutrients and the mucosa. Other procedures are less employed but can be used on selected patients, either in isolation or as a part of combined techniques.

Eventually, multidisciplinary intestinal rehabilitation programs have a key role in promoting intestinal autonomy, favoring the coordination of medical, nutritional, and surgical management. Hepatoprotective PN, optimized enteral support, careful maintenance of central venous lines, new medical and hormonal therapies, such as the GLP-2 analogue, are the first line in treatment of SBS, but in a selected number of patients, AIRS plays a pivotal role in improving enteral tolerance, promoting the achievement of enteral autonomy, increasing the survival of patients, and improving their quality of life.

## Author Contributions

GB and DA: study conception and design. CM, MS, GB, and FP: bibliographic research and literature review. GB and FP: draft manuscript preparation. DA: manuscript revision and correction. GB: drawing pictures. All authors reviewed the results and approved the final version of the manuscript.

## Conflict of Interest

The authors declare that the research was conducted in the absence of any commercial or financial relationships that could be construed as a potential conflict of interest.

## Publisher’s Note

All claims expressed in this article are solely those of the authors and do not necessarily represent those of their affiliated organizations, or those of the publisher, the editors and the reviewers. Any product that may be evaluated in this article, or claim that may be made by its manufacturer, is not guaranteed or endorsed by the publisher.
